# Highlight: A Search for New Species on the High Seas

**DOI:** 10.1093/gbe/evaa156

**Published:** 2020-10-04

**Authors:** Casey McGrath

Out of the vast number of life forms on the planet, it is estimated that fewer than 25% have been characterized, perhaps even fewer than 0.01%. Among this list, microscopic organisms are especially poorly represented: whereas up to 80% of all eukaryotes are protists (i.e., single-celled eukaryotes), these account for only 3% of described eukaryotic species. This striking gap between what we know and what we do not greatly limits our understanding of the evolution of various taxonomic groups and features. In an article in *Genome Biology and Evolution* titled “Gene similarity networks unveil a potential novel unicellular group closely related to animals from the Tara Oceans expedition,” researchers at the Institut de Biologia Evolutiva in Spain and the Institut de Systématique, Évolution, Biodiversité in France shed light on the uncharacterized microbial biodiversity of the world’s oceans and reveal how the discovery of new lineages may aid in addressing unanswered evolutionary questions (Arroyo et al. 2020).

Considerable effort has focused on understanding the evolutionary processes that gave rise to multicellular animals from our unicellular ancestors. For this, researchers have looked to the closest living relatives of animals, a group of protists known as the Holozoa, which includes the Choanoflagellatea, Filasterea, and Teretosporea (Ichthyosporea and Corallochytrea). Previous sampling has suggested that many members of this clade remained uncharacterized, providing an incomplete picture that limits our understanding of the origin of multicellularity. Iñaki Ruiz-Trillo, an ICREA research professor at the Institut de Biologia Evolutiva, and colleagues sought to remedy this by gaining a better understanding of the real diversity of protists closely related to animals.

Environmental metagenomics involves taking samples from the environment—perhaps water from a lake or soil from a forest—and capturing sequences from as many different organisms as possible. Although analysis of the resulting data requires complex methods to group sequences together and links them back to the organisms they derive from, this approach has been successfully used to identify a host of previously uncharacterized organisms. According to Ruiz-Trillo, the ability to “analyze millions of DNA sequences from a small sample of water and know what type of species inhabit that ecosystem is a very powerful technique.”

In their study, the authors analyzed data from over 1,000 samples of ocean water taken from 210 locations around the world. The samples were collected aboard the schooner *Tara*, which crisscrossed the globe as part of a 4-year expedition to sample and characterize planktonic diversity. Because only a small portion of the genome of each species was sequenced, Ruiz-Trillo et al. used gene similarity networks to analyze the data. This technique is generally used to study ecological interactions and has rarely been applied to metagenomic data. It involves drawing webs in which each unique sequence is represented as a point and is connected to similar sequences by a line (fig. 1). Network characteristics such as how connected each point is and which points are in the center versus periphery of the web provide information on the identity and biodiversity of the sequences.

**Figure:**
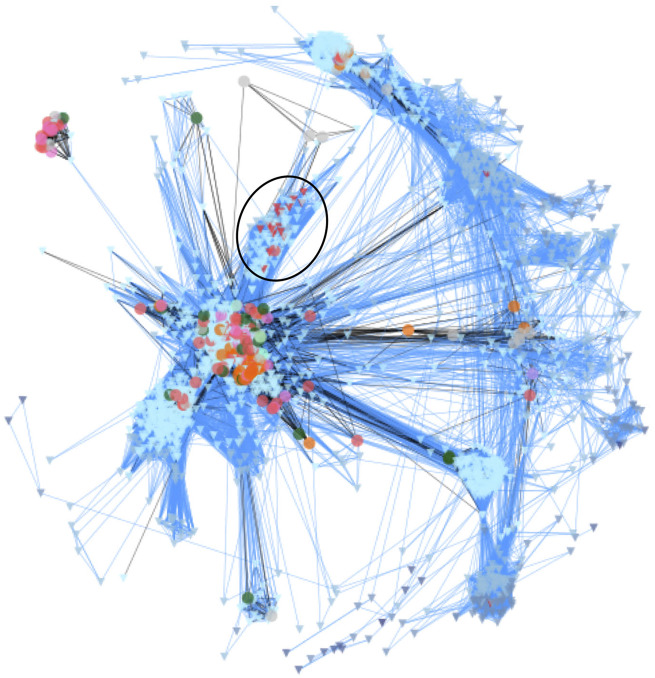
Fig. 1.—Unicellular Holozoa network. Environmental sequences are depicted as triangles, and reference sequences as circles. Lines connect similar sequences. Sequences belonging to the novel Holozoa group MASHOL are shown as red triangles and highlighted in the black circle. Reprinted from Arroyo et al. (2020).

Using this approach, the researchers compared the sequences from the oceanic samples with reference sequences known to originate from members of the Holozoa. Their results revealed over 2,000 unique sequences that are likely to represent unknown/uncharacterized unicellular Holozoans in the world’s oceans. In particular, they identified a group of organisms that are closely related to the choanoflagellates, considered to be the nearest living relatives of animals. Because of this relationship, future analyses of this newly discovered group, referred to by the authors as MASHOL (Marine Small HOLozoa), may provide new insight into the evolution of multicellularity and the origin of animals.

In addition to the discovery of this novel clade, the study provides the first analysis of the geographic distribution of various Holozoans, identifying subgroups that are more abundant in the Arctic, South Pacific, North Pacific, or Atlantic Oceans, as well as those that prefer deeper or shallower water. Finally, the authors looked for Holozoan sequences that co-occurred with specific animal sequences to identify previously unknown interspecies relationships. This analysis provides new hypotheses about which Holozoans may be animal parasites or symbionts.

Although the authors acknowledge that a study based only on sequence data provides a limited perspective, their work contributes to a better understanding of the relationships among different lineages and may pave the way for future studies that identify new microbial species. “Our analyses provide a hint as to where to begin to look for new species or clades. The tree of life is immense, and discovering new microbial species is hard, time-consuming work. Our study suggests habitats where these organisms may be located, as well as what characteristics they might have based on their phylogenetic relationships, providing clues for future attempts at this endeavor.”
